# Comprehensive Assessment of Copy Number Alterations Uncovers Recurrent *AIFM3* and *DLK1* Copy Gain in Medullary Thyroid Carcinoma

**DOI:** 10.3390/cancers13020218

**Published:** 2021-01-09

**Authors:** Aline Neves Araujo, Cléber Pinto Camacho, Thais Biude Mendes, Susan Chow Lindsey, Lais Moraes, Marta Miyazawa, Rosana Delcelo, Renata Pellegrino, Diego Robles Mazzotti, Rui Monteiro de Barros Maciel, Janete Maria Cerutti

**Affiliations:** 1 Genetic Bases of Thyroid Tumors Laboratory, Division of Genetics, Department of Morphology and Genetics, Escola Paulista de Medicina, Universidade Federal de São Paulo, Pedro de Toledo 669, 11 andar, São Paulo 04039-032, Brazil; anaraujo@unifesp.br (A.N.A.); thais.biude@unifesp.br (T.B.M.); lsmoraes@unifesp.br (L.M.); marta.miyazawa@unifal-mg.edu.br (M.M.); 2 Laboratory of Molecular and Translational Endocrinology, Division of Endocrinology, Department of Medicine, Escola Paulista de Medicina, Universidade Federal de São Paulo, Pedro de Toledo 669, 11 andar, São Paulo 04039-032, Brazil; cpcamacho@unifesp.br (C.P.C.); scclindsey@unifesp.br (S.C.L.); Rui.Maciel@unifesp.br (R.M.d.B.M.); 3 Department of Pathology, Escola Paulista de Medicina, Universidade Federal de São Paulo, Rua Botucatu, 740, São Paulo 04023-900, Brazil; rosana.delcelo.ext@dasa.com.br; 4 Center for Applied Genomics, The Children’s Hospital of Philadelphia, Research Institute, 3401 Civic Center Blvd., Philadelphia, PA 191014, USA; silvar@email.chop.edu; 5 Division of Sleep Medicine, Department of Medicine, Perelman School of Medicine, University of Pennsylvania, Philadelphia, PA 191014, USA; diegomaz@pennmedicine.upenn.edu

**Keywords:** medullary thyroid carcinoma, RET, copy number alteration, AIFM3, DLK1

## Abstract

**Simple Summary:**

Medullary thyroid cancer (MTC) is often discovered in its advanced stage. Although a rare disease, advanced MTC cases have poor prognosis and the treatment is often palliative. Several studies have reported the existence of an association between copy number alterations (CNAs) burden and cancer progression. Moreover, the accumulation of broad CNAs, which contribute to intra-tumor heterogeneity, might be required for immune evasion. The identification of the recurrent CNAs associated with tumor phenotype aided in discovering new therapeutics options in several cancer types. To our knowledge, CNA is not well characterized in MTC. We analyzed recurrent focal CNAs on MTC. Our analysis provides a novel insight on MTC biology and may help in uncovering novel potential therapeutic targets.

**Abstract:**

Medullary thyroid carcinoma (MTC) is a malignant tumor originating from thyroid C-cells that can occur either in sporadic (70–80%) or hereditary (20–30%) form. In this study we aimed to identify recurrent copy number alterations (CNA) that might be related to the pathogenesis or progression of MTC. We used Affymetrix SNP array 6.0 on MTC and paired-blood samples to identify CNA using PennCNV and Genotyping Console software. The algorithms identified recurrent copy number gains in chromosomes 15q, 10q, 14q and 22q in MTC, whereas 4q cumulated losses. Coding genes were identified within CNA regions. The quantitative PCR analysis performed in an independent series of MTCs (*n* = 51) confirmed focal recurrent copy number gains encompassing the *DLK1* (14q32.2) and *AIFM3* (22q11.21) genes. Immunohistochemistry confirmed AIFM3 and DLK1 expression in MTC cases, while no expression was found in normal thyroid tissues and few MTC samples were found with normal copy numbers. The functional relevance of CNA was also assessed by in silico analysis. CNA status correlated with protein expression (*DLK1*, *p* = 0.01), tumor size (*DLK1*, *p* = 0.04) and AJCC staging (*AIFM3* *p* = 0.01 and *DLK1* *p* = 0.05). These data provide a novel insight into MTC biology, and suggest a common CNA landscape, regardless of if it is sporadic or hereditary MTC.

## 1. Introduction

Medullary thyroid carcinoma (MTC) is a malignant thyroid tumor originating from the parafollicular C-cells of the thyroid, which accounts for 1–5% of all thyroid cancers worldwide [1]. MTC may occur either in a sporadic (75%) or in a hereditary (25%) form. In the hereditary form it occurs as a component of the type 2 multiple endocrine neoplasia (MEN) syndromes MEN2A (OMIM # 171400) and MEN2B (OMIM # 162300) [1].

Somatic mutations in the rearranged during transfection (*RET*) gene have been described in nearly 50% of sporadic MTCs, while *RAS* mutations occur in about 0–43% of the sporadic cases [2,3]. Germline gain-of-function mutations in the *RET* gene have been associated with inherited predisposition to MEN2A and MEN2B [1,4].

It has been suggested that somatic or germline mutations on the *RET* gene are not the only determinant of the MTC phenotype. In the hereditary form, it represents a favorable genetic background to develop C-cell hyperplasia, which precedes the development of MTC. In fact, in vitro studies with transgenic mice demonstrated that RET-mutant mice displayed several features of human disease, such as bilateral C-cell hyperplasia with progression to bilateral and multicentric MTC, and, eventually, distant metastases to the liver [5]. The classical progression from C-cell hyperplasia to MTC, to loco-regional lymph nodes, and ultimately to distant metastasis, has also been observed in man, and is largely associated with the type of RET mutation [1]. Accordingly, it has been hypothesized that the RET receptor represents the first hit to cause C-cell hyperplasia [1]. Somatic secondary mutations, in one or few preneoplastic C-cells, may confer clonal advantages and tumorigenic properties to the cells.

These somatic genetic changes may include single nucleotide variants (SNVs), small insertions and deletions (indels), structural variants, retrotransposition and copy number alterations (CNA); the latter is defined as somatically acquired gains or losses of a DNA segment in the genome of a population of cells when comparing to a reference genome [6,7]. CNAs can arise during cancer development and progression, and might alter the transcription of genes by altering the dosage or by disrupting the proximal or distal regulatory regions [8,9,10].

While SNVs are more widely studied, few groups have used array comparative genomic hybridization to identify new CNAs potentially involved in the pathogenesis of both hereditary and sporadic MTC. Broad CNAs, which are defined as whole-chromosome- and chromosomal arm-level alterations, have been reported in 1p, 3q, 3p, 4, 7q, 9q, 12p, 13q, 19 and 22q in both sporadic and germline RET-positive MTC [11,12,13,14,15,16,17,18]. Losses of 1p, 4q and 22q were the most common changes found in MTC. The loss of chromosome 22 occurred more frequently in sporadic MTC [15].

According to the proposed models, as a tumor grows it acquires extra CNA regions, some of which are tumor-specific [19]. The accumulation of broad CNA, which contribute to intra-tumor heterogeneity, might be required for tumor development, progression and immune evasion, and may confer a high risk of lethal diseases. Importantly, tumor heterogeneity contributes to the development of drug resistance and, therefore, might represent a novel target for therapy. Although several broad CNAs have been identified in MTC, none of the identified alterations have been shown to play a significant role in the development or progression of MTC. In fact, the precise role of focal CNAs, which are alterations of limited size, in tumor initiation and progression, as well as their clinical relevance, is still poorly understood.

In the present study, we used the Affymetrix genome-wide human SNP array 6.0 in MTC and paired blood samples to identify recurrent focal CNAs that contain entire genes and, therefore, directly affect the expressions of the genes on the altered genomic segment. 

We believe that the CNA profile identified in this study brings insights into the pathogenesis of MTC, as cancer-specific copy number gain correlates with increased protein expression. Whether the acquired CNAs identified in this study might improve disease prognostication, follow-up or even targets for drug development still remains to be determined.

## 2. Results

### 2.1. Identification of CNAs in MTC Genome

**Figure 1:**
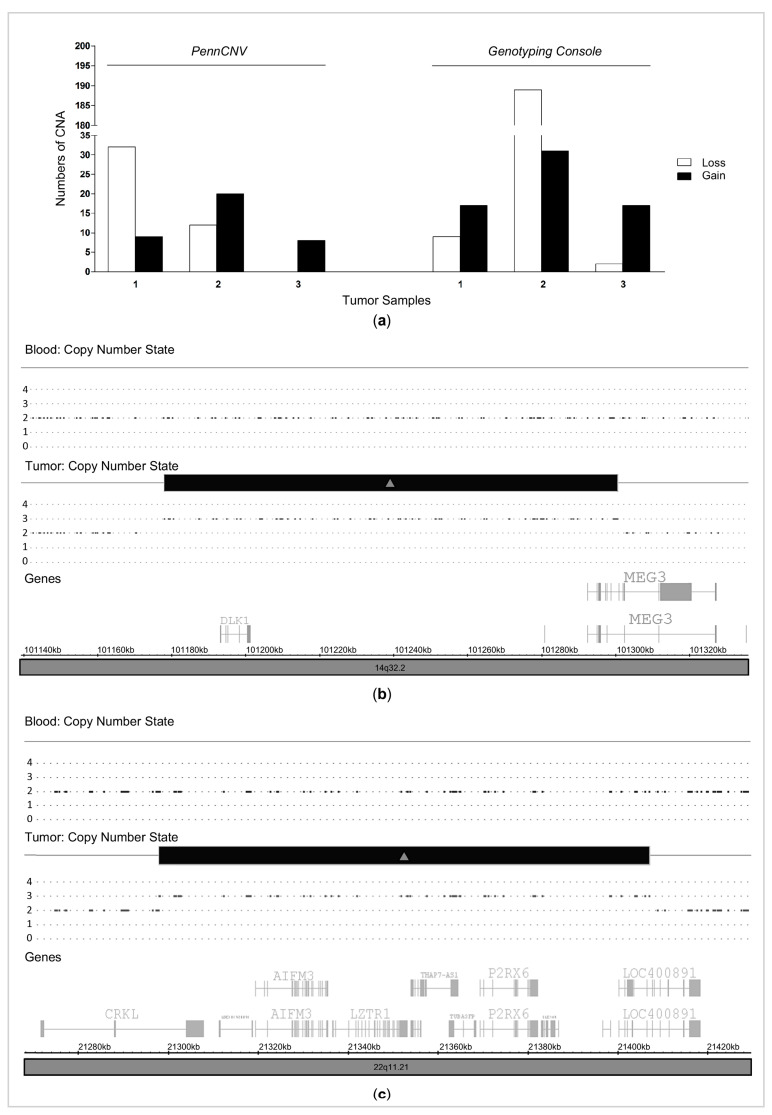
Identification of CNAs in MTC Genome using both PennCNV and Genotyping Console software. (**a**) Distribution of copy number alterations in MTC samples. (**b**) An illustration representing a patient’s data (case 3) for a candidate CNA gain (present on tumor and absent on paired blood) on 14q32.2. (**c**) An illustration representing a patient’s data (case 2) for a candidate CNA gain (present on tumor and absent on paired blood) on 22q11.21. The Log2 ratio represents the normalized intensity related to a reference (diploid) genome. The copy number state (HMM derived) is based on Log2.

Genome-wide analysis was performed in a discovery cohort that consisted of matched MTC tissue and blood samples from three patients. The analysis was conducted using both PennCNV (NCBI36/hg18) and Genotyping Console (GRCh37/hg19) software. A comprehensive analysis of the CNAs detected by the two software packages in the MTC samples, which are absent in matched blood, is shown in Table 1. Genotyping Console detected higher numbers of, and larger, CNAs (266 CNAs, mean size 279 kb) than PennCNV (81 CNAs, mean size 43 kb (Figure 1a; Table S1)). PennCNV analysis revealed that MTC samples presented from 8 to 41 CNAs, while Genotyping Console uncovered that MTC samples presented 19 to 220 CNAs. Although CNAs were observed across all chromosomes, a high number were distributed on chromosomes 1, 4 and 8.

To identify the CNAs that are more likely associated with a more general tumor phenotype and to avoid intra-tumor heterogeneity, we selected those CNAs that met the minimum criteria; i.e., segments greater than 1000 bp that were found in at least two cases (out of three total cases) but were absent in matched controls (three peripheral blood samples). Although the sample size is small, we identified seven regions that met this criterion, i.e., six recurrent regions with focal gains and one recurrent region with focal loss. The regions as well as identified genes are presented in Table 1.

### 2.2. Recurrent Gains in Medullary Thyroid Carcinoma

Overall, the total number of losses significantly exceeded the total number of gains and amplifications (Figure 1a; Table S1). However, when we evaluated those CNA that met the minimum criteria, recurrent gains (9p12, 10q26.3, 14q32.2, 15q11.1, 15q11.2, 22q11.21) were more common than losses (4q28.3). Because telomeric and centromeric regions are likely to harbor spurious CNA calls, CNAs located near a centromeric region (15q11.1) were not considered for the validations steps. Essentially, genes were mapped within five regions (15q11.2, 9p12, 10q26.3, 14q.32.2 and 22q11.21) that showed copy number gains (Table 1). As the segment with CNAs includes whole or partial gene amplification/deletion, we selected a protein-coding gene that was entirely located within each CNA to validate the copy number state by quantitative PCR (Figure 1b,c, Table 1). This strategy would enable cancer gene discovery.

The regions comprising the genes *SNORD116-18 (*15q11.2)*, FGF7P3 (9p12), ADAM8 (*10q26.3)*, DLK1* (14q.32.2) and *AIFM3* (22q11.21), and the region containing no gene (4q28.3), were experimentally validated by quantitative PCR (qPCR) in the discovery cohort. The experimental analysis confirmed copy number gain in MTC samples as compared to their paired blood sample for the three regions encompassing the genes *SNORD116-18, DLK1* and *AIFM3*. The other three regions, comprising the genes *FGF7P3* and *ADAM8* or containing no gene, were not validated in this initial validation step, and therefore, were excluded from further analysis.

**Figure 2:**
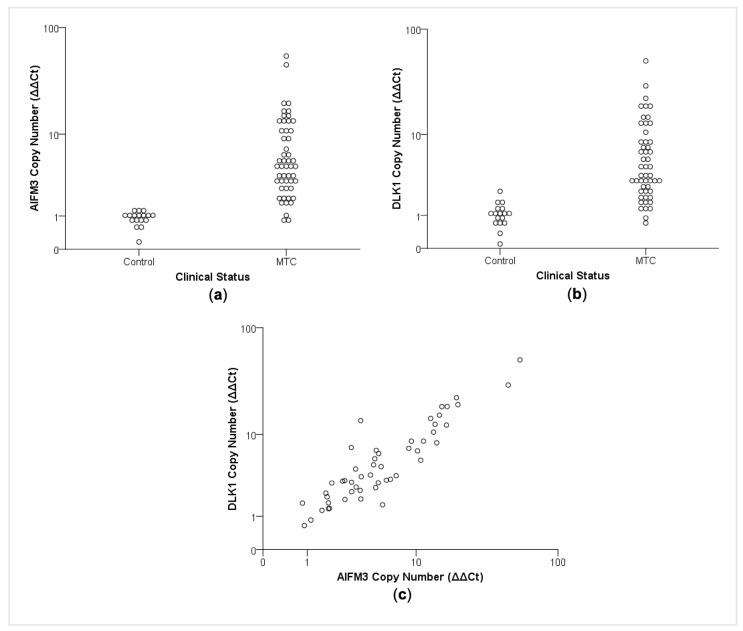
Experimental analysis of recurrent CNA gains in an independent validation cohort. Recurrent gain of (**a**) *AIFM3* and (**b**) *DLK1* was observed in most MTC samples, while they were not observed in non-matched blood samples (control) (*p* < 0.01; Mann–Whitney test). (**c**) Positive correlation between *AIFM3* and *DLK1* copy number (r_s_0.55, *p* < 0.01).

As the next step, the recurrent copy number gains were experimentally tested by qPCR on an independent validation cohort that included 51 MTC tissues and 17 non-matched blood samples (control group). Using this completely independent data set, we confirmed the copy number gain in the region containing the *AIFM3* (*p* < 0.01) and *DLK1* (*p* < 0.01) genes for most MTC samples, compared to the control group (Figure 2a,b). Remarkably, we found a positive correlation between *AIFM3* and *DLK1* copy number (r_s_0.55, *p* < 0.01) in this independent set of MTC (Figure 2c).

The focal copy number gain, which includes the *SNORD116-18* gene (15q11.2), was not confirmed in the validation cohort, and, therefore, was not included in the further analysis.

### 2.3. CNA Affects AIFM3 and DLK1 Expression in Medullary Thyroid Carcinomas

As the duplication of an entire gene can lead to an increased number of functional copies, i.e., increased gene dosage, we assessed the impact of *AIFM3* and *DLK1* copy-number gain on protein expression.

**Figure 3:**
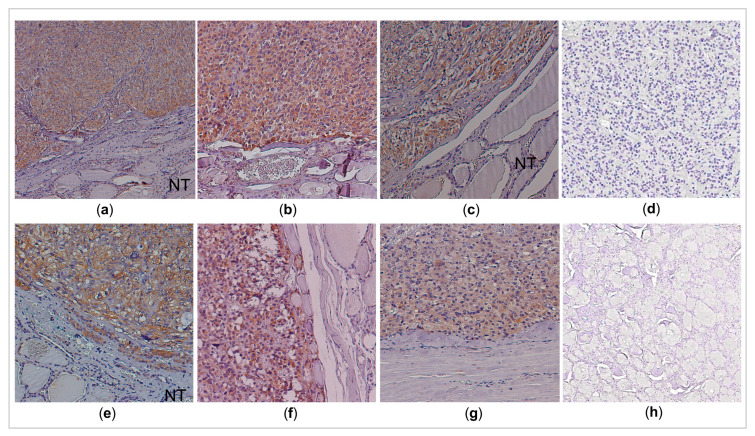
Representative immunohistochemistry results of AIFM3 and DLK1 in medullary thyroid carcinomas samples. Most medullary thyroid carcinomas samples showed positive staining for (**a–c**) AIFM3 and (**e–g**) DLK1, while their expressions were not detected in very few (**d**) medullary thyroid carcinomas (**h**) and in the adjacent normal thyroid. Original magnification ×200. NT, normal thyroid.

The immunohistochemistry analysis was performed in 27 out of 51 MTC cases for which paraffin-embedded sections were available (Table S2). Briefly, AIFM3 staining was observed in the majority of the MTC cases (24/27; 89%). Most of the AIFM3 positive cases showed either strong (*n* = 11) or moderately abundant (*n* = 12) staining. DLK1 staining was seen in 93% of the MTC cases (25/27). Strong (*n* = 14) or moderately abundant (*n* = 9) staining was observed in the majority of the samples. Interestingly, when a tumor sample showed a negative or weak staining for AIFM3, it also showed a similar pattern for DLK1 (*p* < 0.02) (Figure 3). Importantly, AIFM3 and DLK1 proteins were not detected in the normal thyroid tissues adjacent to the positive carcinoma tissues (Figure 3), confirming that they are cancer-related genes.

**Figure 4:**
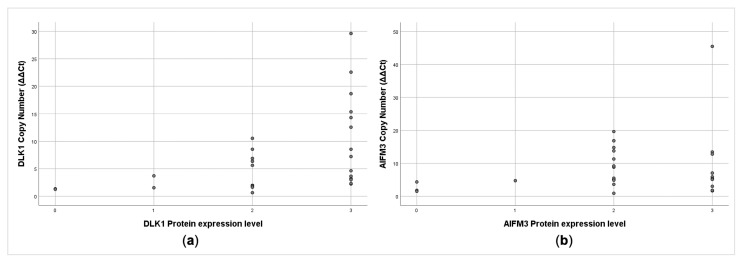
Correlation between genes copy numbers and their protein levels (**a**) *DLK1* (r_s_ = 0.62, *p* = 0.01). (**b**) *AIFM3* (r_s_ = 0.13, *p* = 0.51).

Significantly, the copy number gain of *DLK1* positively correlated with DLK1 protein expression (rs = 0.62, *p* = 0.01; Figure 4a). No correlation was found between *AIFM3* copy number gain and AIFM3 protein expression (r_s_ = 0.13, *p* = 0.51, Figure 4b).

Collectively, these analyses suggest that CNAs have an impact of on gene and protein expression, indicating that these copies are likely functional. However, functional analysis is needed to better understand the roles of AIFM3 and DLK1 in MTC.

### 2.4. Correlations between CNA and RET Mutational Status

**Figure 5:**
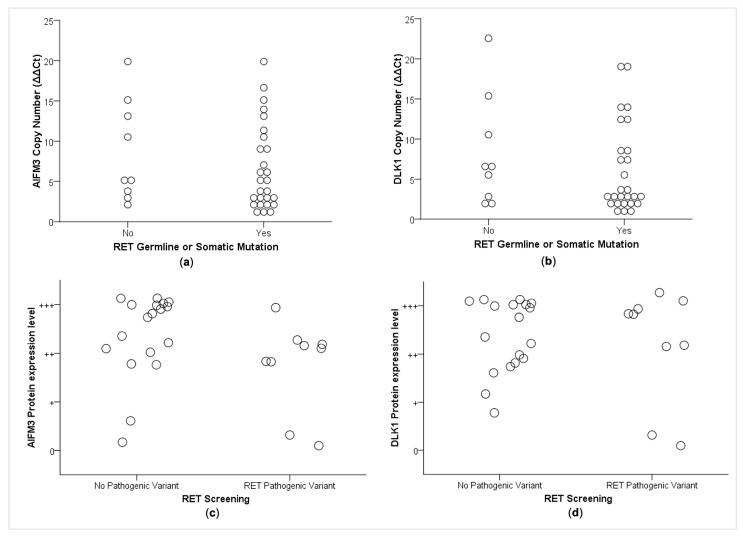
Association between *RET* mutational status and CNA of the (**a**) *AIFM3* (*p* > 0.05) and (**b**) *DLK1* (*p* = 0.56) genes. The relative copy number state was calculated using the 2^(−ΔΔCt)^, and copy number gain was defined as ΔΔCt above 1.5. Association between *RET* mutational status and protein expression level of the (**c**) *AIFM3* (*p* = 0.58) and (**d**) *DLK1* (*p* = 0.58). +++ Strong staining. ++ Moderately abundant staining. + Weak staining. 0 Negative.

The *RET* mutational status (presence of somatic or germline *RET* variant) was associated with neither the CNA status of the *AIFM3* and *DLK1* genes (Figure 5a,b) nor with their protein expression (*p* > 0.05, data not shown). Considering only its inherited pattern, the presence of a germline *RET* variant was also associated with neither CNAs of AIFM3 (*p* > 0.05) or DLK1 (*p* = 0.29) nor with their protein expression (Figure 5c,d).

### 2.5. AIFM3 and DLK1 Copy Number Alteration was Associated with Clinical–Pathological Parameters

We next tested whether there is an association between a higher *AIFM3* and *DLK1* copy number state and age of onset, gender, tumor size, multifocality, extrathyroidal extension, vascular invasion and AJCC staging system.

**Figure 6:**
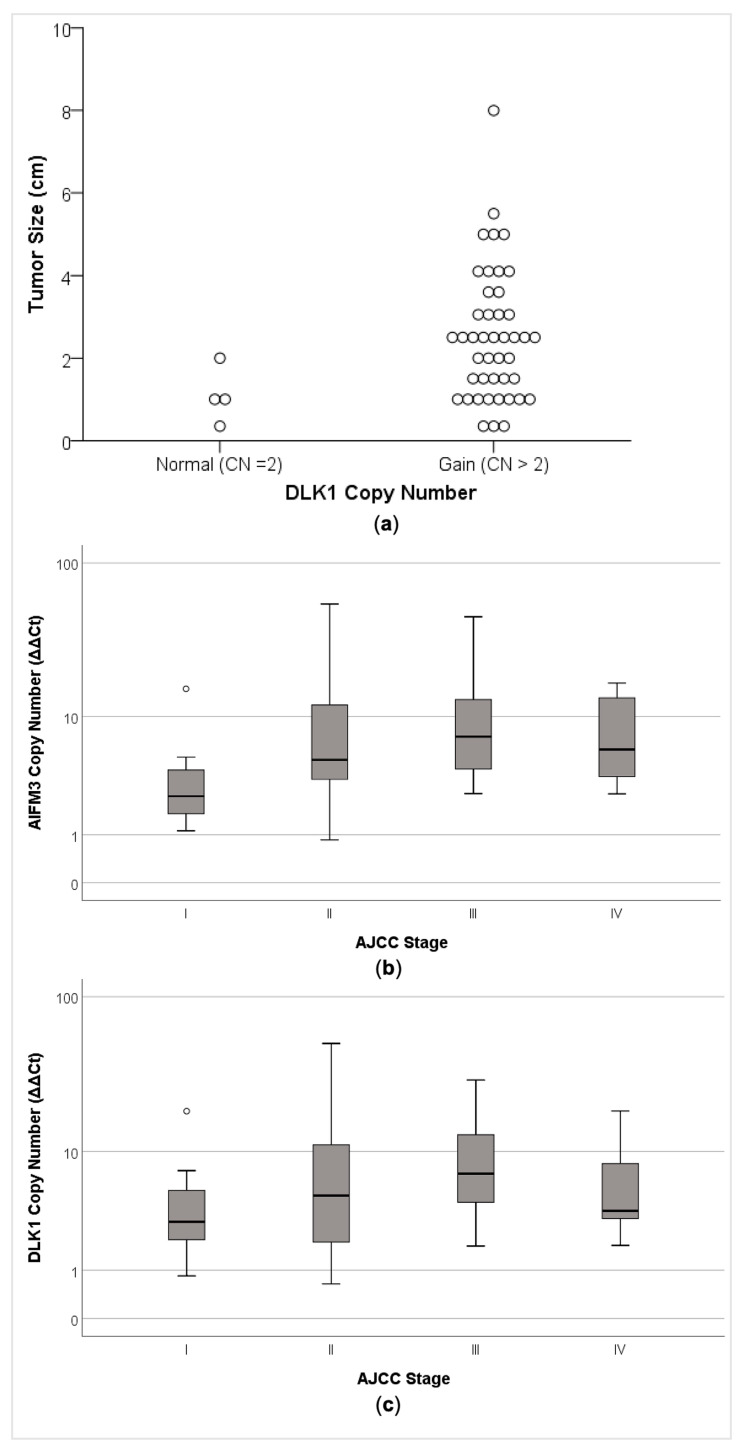
Association between *AIFM3* and *DLK1* copy number alteration with clinical–pathological parameters. (**a**) Association of copy number gain of *DLK1* gene and larger tumor size (*p* = 0.037; Mann–Whitney test). (**b**) Association between AJCC stage and the copy number of the *AIFM3* (r_s_ = 0.39, *p* = 0.01) and (**c**) *DLK1* (r_s_ = 0.29, *p* = 0.05). The relative copy number state was calculated using the 2^(−ΔΔCt)^, and copy number gain was defined as ΔΔCt above 1.5.

Although no association was found between DLK1 copy number and age (*p* = 0.86), gender (*p* = 0.55), multifocality (*p* = 0.55), extrathyroidal extension (*p* = 0.68) and vascular invasion (*p* = 0.24), *DLK1* copy number gain was associated with a larger tumor size (*p* = 0.04; Figure 6a).

AIFM3 copy number gain was not associated with age (*p* = 0.73), gender (*p* = 0.26), tumor size (*p* = 0.45), multifocality (*p* = 0.21), extrathyroidal extension (*p* = 0.78), vascular invasion (*p* = 0.49) or pTNM (*p* = 0.24).

Remarkably, *AIFM3* (r_s_ = 0.39, *p* = 0.01) and *DLK1* copy number gain (r_s_ = 0.29, *p* = 0.05) correlated with a worse AJCC stage (III and IV) (Figure 6b,c).

### 2.6. AIFM3 and DLK1 Protein Expression and Clinical–Pathological Parameters

No correlation was found between clinical–pathological parameters and *DLK1* and AIFM3 protein expression.

## 3. Discussion

Although RET (rearranged during transfection) is the bona fide driver oncogene in hereditary medullary thyroid carcinoma, it has been suggested that the germline mutation of the cancer-predisposing gene does not trigger cancer *per se*. In other words, a fraction of the total cells that have the founder genetic event holds the genetic background to develop C-cell hyperplasia [1]. The pre-malignant cells may evolve into MTC in the presence of additional somatic mutations or epigenetic events that give competitive advantages over cells that have not acquired novel genetic events [5]. Moreover, the progression to a more invasive MTC, to loco-regional lymph nodes and ultimately to distant metastasis is likely associated with additional driver mutations. Therefore, the development and progression of MTC is seemingly more related to the accumulation of genomic aberrations, such as deletions, gains and fusion gene events, than to more localized mutational events.

In the past 10 years, several studies have used array comparative genomic hybridization (aCGH), high-density SNP arrays and next generation platforms to identify focused mutations, small insertions and deletions (indels), copy number alterations (CNA) and large structural alterations in sporadic and hereditary MTC. Although multiple somatic events have also been reported in sporadic MTC, which have been associated with clinical outcome [20], none of these studies have identified recurrent genetic alterations that were associated with MTC development and progression. Our group has recently identified somatic-specific RET retroposed copies in MTC samples and thyroid medullary carcinoma cell lines (TT and MZ-CR-1) [21] that may occur as a second hit.

In the present study, genome-wide copy number analysis was performed in MTC and paired blood samples to identify recurrent CNA in MTC samples.

To measure the specificity of CNA detection and to increase the probability of identifying recurrent CNA, the validation analysis was performed using a two-step process. We initially selected CNAs that were present in at least two out of three MTCs and were absent in paired blood samples, and performed experimental validations in the discovery cohort. This strategy would reduce the incidence of false-positive results.

As CNAs are somatic changes that result in the gain or loss of segments of DNA that might contain genes, we primarily investigated the effects of CNA on protein-coding genes. Consequently, we chose genes that were entirely located within the variable region to validate the copy number state. Although an inappropriate sample size can lead to error, the experimental validation in the discovery cohort confirmed the copy-number gain for three selected genes (*SNORD116-18*, AIFM3 and *DLK1*).

Next, the recurrent copy number gains were experimentally validated in an independent set of 51 MTC samples and controls. The copy number gain was confirmed in most MTC samples for both *AIFM3* and *DLK1*, as compared to non-matched blood controls.

It is well known that structural variations in the genome, such as CNA, can influence gene expression through different mechanisms [22]. These observations triggered the question of what the consequences are of these dominantly selected CNAs with respect to the translation level of genes on this genomic segment. Therefore, we investigated whether copy number gains of *AIFM3* and *DLK1* affected the protein expression of these genes.

Although in the presence of somatic copy number change we do not expect a linear correlation between copy number, mRNA or mRNA and protein expressions for all affected genes [23], the AIFM3 and DLK1 proteins’ expression increased in most MTC tissues, while their expression was absent in normal thyroid. As such, even if a change in DNA copy number is just one mechanism associated with changes in gene expression, the expression analysis allows us to demonstrate that the *DLK1* copy number gain was positively correlated with protein expression, even in a nonlinear way.

All together, these findings suggest that the recurrent genetic alterations found in this study are likely functional, and may be associated with MTC development and progression.

Apoptosis-inducing factor mitochondrion-associated 3 (AIFM3) is a predicted intracellular protein (mitochondria), where metal ion binding (Gene ontology, GO) is highly conserved and known to induce cell death via mitochondrial depolarization, cytochrome C release and caspase-3 activation [24].

The functional analysis performed using databases and enrichment analysis confirmed AIFM3 copy number gain and gene overexpression in several cancer subtypes (Catalogue of Somatic Mutations in Cancer (COSMIC), Table S3), while very low expression levels have been observed in several normal tissues, including thyroid, adrenal, prostate, liver, lung, and others (GTEX, FANTOM5 and Human Protein Atlas dataset).

Its role in cancer is controversial. It has been suggested that *AIFM*3 expression is a direct target of miR-210 [25,26], whereas the down-regulation of miR-210 and the increased expression of *AIFM3* inhibits proliferation, induces apoptosis and enhances radiosensitivity in hypoxic human hepatoma cells in vitro [25]. Instead, the overexpression of AIFM3 was found in the mitochondria of cholangiocarcinoma tissues, but not in the adjacent non-cancerous tissues [27]. To better understand the role of AIFM3 overexpression in cholangiocarcinoma, the authors explored the protein–chemical interaction networks and found that AIFM3 linked to various key molecules in cancer progression through z-VAD-fmk, which is a pan-caspase inhibitor widely used to block apoptosis [27]. AIFM3-related proteins predicted by STITCH showed that AIFM3 was associated with superoxide dismutase 2 (SOD2), NADPH oxidase 1 (NOX1), serine hydroxymethyltransferase 2 (SHMT2) and protein kinase C alpha PRKCA [27]. Recently, AIFM3 was found to be overexpressed in breast cancer patients and was significantly associated with tumor size, lymph node metastases, TNM staging, and a shorter overall survival and disease-free survival [28]. Similarly, we here found an important correlation between *AIFM3* copy number gain, AJCC III and IV stage.

Delta-like non-canonical Notch ligand 1 (DLK1), aliases DLK, Pref-1 and pG2, encoded a transmembrane glycoprotein that contains multiple epidermal growth factor repeats [29]. DLK1 is widely expressed during organism development [29], and its expression diminishes along with increased cell differentiation. Few groups have identified DLK1 as a candidate stem/progenitor cell marker. In adults, few tissues retain the expression of DLK1, such as brain, but this is re-expressed throughout diseases and regeneration. The functional analysis revealed *DLK1* copy number gains and gene overexpression in several cancer subtypes (Catalogue of Somatic Mutations in Cancer (COSMIC), Table S3).

The deregulation of the imprinted DLK1-DIO3 locus at 14q32.2–32.31 has been associated with replicative senescence, stem cell function and cancer [30]. Interestingly, an increased expression of DLK1 and DIO3 was observed in a large intrathoracic tumor of a patient who developed consumptive hypothyroidism. The authors showed that the increased expressions of DIO3 and DLK1 likely occurred through SHH and/or MAPK pathways [31]. Others have also suggested that the MAPK and SHH pathways modulate DIO3 expression in papillary thyroid carcinomas and in an MTC cell line [32]. Recently, it was demonstrated that the osteogenic fate of human pluripotent stem cell lines is associated with the methylation status and the expression of miRNAs from the imprinted *DLK1/DIO3* locus [33].

Remarkably, DLK1 is involved in human cancer. It was found to be expressed in tumors with neuroendocrine features, such as neuroblastoma, pheochromocytoma, and a subset of small cell lung carcinoma cell lines. In normal tissues, DLK1 expression was restricted to the adrenal gland and placenta. Therefore, the authors suggested that DLK1 might be involved in neuroendocrine differentiation, and could be a potential therapeutic target in neuroendocrine tumors [34]. They also proposed that DLK1 is a useful immunohistochemical marker for the identification of adrenocortical tumors [35]. Recently, it was demonstrated that DLK1 promotes tumorigenesis and the epithelial–mesenchymal transition of ovarian high-grade serous carcinoma through the activation of Notch signaling [36].

We here suggest that one additional mechanism associated with DLK1’s increased expression in MTC is copy number gain. Database retrieval revealed that the copy number gain of *DLK1* was associated with pathogenic phenotypes. DLK1 seems to be associated with cell proliferation, and was also found to be an unfavorable prognostic marker in endometrial cancer (https://www.proteinatlas.org/search/DLK1).

Importantly, our current findings should be interpreted within the context of some potential limitations. Nearly 50 genes were targeted in the regions affected by CNA in the MTC samples (Table 1). It is hard to discriminate which gene placed within a recurrently deleted or gained/amplified region is a real cancer driver. Therefore, we cannot exclude the possibility that the remaining genes, in the same region, that we have not validated, also have an impact in the pathogenesis of MTC. Moreover, the biological consequences might not be limited to the expression of the genes present on the altered segment. The segment may contain regulatory elements that affect the transcriptional activity of genes residing in other areas of the genome.

Ultimately, it has been recognized that CNAs are specific to the tissue of cancer origin and, therefore, reflect specific sceneries of genomic imbalances for each tumor. Therefore, to better define the roles of DLK1 and AIFM3 in the progressions of MTC is essential.

## 4. Materials and Methods

### 4.1. Subjects

Frozen section specimens from MTC tissues and matched blood samples collected from 3 patients who underwent thyroidectomy at Hospital São Paulo, Universidade Federal de São Paulo, Brazil, were used as the discovery cohort (Table S4). For the experimental validation analysis, an independent set of 51 archived formalin-fixed paraffin-embedded (FFPE) sections was obtained from patients who underwent thyroidectomy during the period from 2001 to 2015 at the same hospital. In order to confirm the diagnosis of MTC and to define the percentage of tumor cells in each sample, hematoxylin and eosin-stained sections were retrieved and reviewed by an experienced pathologist (RD). The demographic and clinical-pathological features of the validation cohort, such as age at diagnosis, gender, tumor size, multifocality, extrathyroidal extension, vascular invasion, presence of lymph node metastasis and *RET* mutational status, are summarized in Table S5. Peripheral blood samples from 17 healthy individuals without a known history of thyroid disease were used in this study as the control group. The study was approved by the Institutional Ethical Review Board of Universidade Federal de São Paulo (nº. 569.937).

### 4.2. DNA Isolation

DNA from peripheral blood was extracted using a standard phenol/chloroform method, as described previously [37]. For formalin-fixed and paraffin-embedded (FFPE) sections, a representative tumor block with the highest percentage of tumor content was selected. Samples containing a percentage of tumor cells greater than 70% were subjected to DNA isolation. Samples containing less than 70% tumor cells were manually macrodissected to enrich the proportion of neoplastic cells, and were subsequently subjected to DNA isolation. The DNA from either the FFPE sections or the fresh-frozen tissues was isolated using the NucleoSpin Tissue Kit (Macherey-Nagel GmbH & Co. KG, Düren, Germany) according to the instructions. DNA samples were quantified using a NanoDrop spectrophotometer (Thermo Fisher Scientific Inc, Waltham, MA, USA).

### 4.3. RET Mutation Analysis

Germline mutations in the exons 8, 10, 11, 13, 14, 15 and 16 were previously investigated as part of the Brazilian Genetic Screening Program, the BrasMEN study [38]. For this study, somatic mutations in exons 10, 11 and 16 of the *RET* gene were investigated in sporadic cases whose DNA was available for *RET* screening analysis (Table S5). About 150 ng of DNA was amplified in a 25 μL PCR reaction containing 5 pmol of each specific primer, 1X PCR Buffer, 0.2 mM of each dNTP, 1.5 mM MgCl*2* and 2U Taq DNA polymerase. The PCR products were purified using the Illustra GFX PCR purification kit (GE Healthcare Corp., Piscataway, NJ, USA), according to instructions. Purified products were sequenced using a BigDye terminator on an ABI 3130 DNA Analyzer (PE Applied Biosystems, Foster City, CA, USA). Each sample was sequenced at least twice and in both directions.

### 4.4. Copy Number Analysis

To identify somatic CNAs that may contribute to disease phenotype, genome-wide DNA copy number analysis was performed in the discovery cohort. The samples were genotyped using the Affymetrix Genome-Wide Human SNP Array 6.0 genotyping arrays (Affy6) (Affymetrix Inc, Santa Clara, CA, USA) according to the recommendations. This array included probes for the detection of over 909,600 SNPs and an additional 946,000 non-polymorphic oligonucleotides for the assessment of copy number variation (CNV). The average of the inter-markers distance is <700 bp. To measure a quantitative locus-level copy number estimate (CNE) for each of the tumor and blood samples, raw files were preprocessed to extract signal intensity for SNP or CNV probe to log_2_ R ratio and B-allele frequency values. Regions of CNA were identified using a Hidden Markov Model (HMM) algorithm provided within PennCNV [39]. To minimize the impact of type I errors, regions containing CNA were defined as those spanning at least three or more consecutive SNPs per segment. We additionally used Genotyping Console 4.1 (Affymetrix Inc.) to analyze the data from Affy6 array, according to the description. The log_2_ ratio and allele difference values were used as the input for Genotyping Console 4.1 software. Genotyping calls were generated using the Birdseed V2 algorithm and the calculation of the copy number (CN) state was established using the Canary algorithm.

The CN changes (gains or losses) of each sample were determined after normalization against the HapMap samples of The International HapMap Consortium [7]. The values of CN were determined using the HMM model, which is based on the log_2_ ratio intensity and B-allele frequency data and estimates up to five copy number states as follows: homozygous deletion (CN = 0), hemizygous deletion or single copy loss (CN = 1), neutral copy number or normal diploid (CN = 2), single copy gain (CN = 3), and gain ≥ 2 copies (CN = 4).

In order to identify cancer-specific alterations that are likely to be pathogenic, CNAs were selected based on the following minimum criteria: (1) segments length greater than 1000 bp; (2) absent in DNA from the matched peripheral blood; (3) present in at least 2 out of 3 MTC samples tested.

### 4.5. Recurrent CNAs were Experimentally Validated by Quantitative PCR

CNAs that met the minimum criteria were validated by quantitative PCR (qPCR). If a CNA event occurred in a region containing at least one gene, primers were designed to target the gene region and its adjacent areas.

The first step of validation was performed in the 3 MTC samples used for initial screening (case) and in the matched peripheral blood samples (control). The second step of the experimental validation was performed in the DNA isolated from an independent set of 51 MTC specimens (case group) and 17 non-matched peripheral blood samples (control).

qPCR was performed in a 12 μL PCR reaction containing 30 ng of DNA, 0.2 μM of each specific primer and 1X SYBR Green PCR Master Mix (Applied Biosystems Foster City, CA, USA). qPCR reactions were run on an Applied Biosystems 7500 Real-Time PCR System (Applied Biosystems, Foster City, CA, USA). The primers were designed to amplify a region within the deleted/duplicated region of interest. *ACTB* was used as the endogenous reference gene, as no copy number variation was reported in peripheral blood from normal individuals. The primer sequences are listed in Table S6. Amplification efficiency was estimated for each set of primers. The specificity of the PCR amplifications was checked by both gel electrophoresis and melting curve analysis. Each sample was analyzed in triplicates. The relative copy number state was calculated using the 2^(−ΔΔCt)^ method, where ΔΔCt = (Ct _target region on case_ − Ct _reference gene on case_)/(Ct _target region on control_ − Ct _reference gene on control_). Copy numbers below 0.5 and above 1.5 were defined as lost/deleted and gained/amplified, respectively.

### 4.6. Association of CNA with Protein Expression

As focal CNA regions overlapped with genomic regions that encompass entire genes, we tested whether the copy number status (loss, no-change, gain) observed had dosage effects on its own protein expression levels across tumors. Immunohistochemistry (IHC) was performed on available paraffin-embedded sections from MTC samples whose tumors were previously tested for CNA (*n* = 27). Primary rabbit polyclonal antibodies specific for AIFM3 (ab106359; Abcam, Cambridge, UK) and DLK1 (ab21682; Abcam) were used. Sections (4 μm) were deparaffinized in xylene and rehydrated through a series of graded ethanols. The endogenous peroxidase activity was blocked by 15% hydrogen peroxide. After steaming retrieval (AIFM3—10 mM Tris-EDTA, 0.5% Tween, pH 9.0, for 15 min; DLK1—10 mM citrate buffer, pH 6.0, for 10 min), the sections were allowed to cool down for 1 h, washed twice with PBS and blocked with PBS/BSA 1% for 30 min at room temperature. Slides were then incubated with primary antibody in a humid chamber for at least 16 h at 4 °C. AIFM3 and DLK1 antibodies were dilute in PBS/BSA 1% at 1:400 and 1:750 dilution, respectively. The labeled DAKO EnVision+ Dual Link System-HRP (DAKO, Carpinteria, CA, USA) and substrate 3,3’-diaminobenzidine tetrahydrochloride (DAB) (DAKO, Carpinteria, CA, USA) were used for immunodetection. Hematoxylin was used as the nuclear counterstain. Positive and negative controls were used in each run. AIFM3 and DLK1 staining were evaluated manually using light microscopy. The grade of staining was defined in a semiquantitative manner as follows: –, negative; +, weak; ++, moderately abundant; +++, strong [40].

### 4.7. Correlations of CNA Alteration and Protein Expression with RET Status, Demographic and Clinical–Pathological Parameters

We investigated an association between CNAs and their own protein expression with data on RET mutational status (non-mutated vs. mutated), inheritance pattern (sporadic or hereditary), demographic (age of onset, gender) and pathological features (tumor size, presence of capsule, capsular invasion, extrathyroidal extension, multifocality, vascular invasion, pTNM and tumor staging), recorded followed the AJCC staging system (8th edition) (Table S5).

### 4.8. Functional Annotation of Identified CNAs and Enrichment Analysis

To better understand the functional impact of the recurrent CNAs identified in this study, we used publically available databases, such as the database of genomic variation and phenotype in humans using ensemble resources—DECIPHER (https://decipher.sanger.ac.uk), ClinVar (https://www.ncbi.nlm.nih.gov/clinvar), Catalogue of Somatic Mutations in Cancer—COSMIC (http://cancer.sanger.ac.uk/cosmic), Genotype-Tissue Expression—GTEx (https://www.gtexportal.org/home), FANTOM5 (http://fantom.gsc.riken.jp), Human Protein Atlas—HPA (https://www.proteinatlas.org) and Enrichr (http://amp.pharm.mssm.edu/Enrichr/enrich). For the classification of the variants, we used the five-tiers system (benign, likely benign, uncertain significance, likely pathogenic and pathogenic), as recommended by The American College of Medical Genetics and Genomics (ACMG) [41]. We additionally uploaded the gene names/symbols to AmiGO 2 (http://amigo.geneontology.org) searching for overrepresentation in Gene Ontology (GO) classification, such as the biological process, molecular function and cellular component.

### 4.9. Statistical Analysis

Values are expressed as mean ± standard error of the mean (SEM). Continuous variables were tested for normality by the Shapiro–Wilk test. To analyze the values of the continuous variables, we performed either the two-tailed Mann–Whitney *U* test or the Unpaired *t*-test. By the use of ordinal variables, we performed the correlation analysis using a nonparametric Spearman’s *ρ* correlation test. Even so, to provide a better representation of the correlation, a nonlinear regression model was used to construct the regression line on the plots. The continuous or ordinal variables were categorized using an ROC curve with a Youden method. We used Fisher’s exact test to analyze categorical or categorized data. The comparison between more than two groups was performed using the Kruskal–Wallis test. A *p*-value of ≤ 0.05 was considered statistically significant. Statistical analysis was performed with IBM SPSS Statistics 26 (IBM Corp. in Armonk, NY, USA).

## 5. Conclusions

In summary, in this study we explored the possible genetic mechanism of CNA in the development and progression of MTC. We also showed that combining CNA and gene expression analysis increased our ability to identify likely drivers in MTC. Further analysis is needed to understand the mechanisms involved in the gain/amplification of the copies of these genes, and to decipher the temporal sequence of the events as MTC develops and progresses. The translational significance of CNA requires the defining of whether the CNAs observed are MTC-specific, and if they will help to improve disease prognostication and follow-up, or even guide therapeutic strategies.

## Figures and Tables

**Table 1:** Copy number alterations found in the discovery cohort.

Software	MTC * Sample	Chr	Start Position **	End Position	Markers	Length (kb)	CN	CN	Genes
**PennCNV**	2, 3	15q11.2	22849641	22885568	37	36	4	gain	*SNORD116-2, SNORD116-3, SNORD116-4, SNORD116-5, SNORD116-6, SNORD116-7, SNORD116-8, SNORD116-9, SNORD116-10, SNORD116-11, SNORD116-12, SNORD116-13, SNORD116-14, SNORD116-15, SNORD116-16, SNORD116-17, **SNORD116-18**, SNORD116-19, SNORD116-20, SNORD116-21.*
**Genotyping Console**	1, 2	4q28.3	137428273	137528519	67	100	1	loss	none
		15q11.1	20130129	20291301	30	161	3	gain	none ***
	1, 3	9p12	41475094	44244868	84	2770	3	gain	*SPATA31A5, SPATA31A7, FAM74A1, FAM74A6, ZNF658B, GLIDR, **FGF7P3**, ANKRD20A2, ANKRD20A3, GXYLT1P3, FAM95B1, FOXD4L4, AQP7P3, FAM74A7, SPATA31A6, CNTNAP3B, CNTNAP3P2.*
	2, 3	10q26.3	135076596	135184421	36	108	3	gain	***ADAM8*** *, TUBGCP2, ZNF511, CALY, PRAP1, FUOM, ECHS1.*
		14q32.2	101178067	101300567	75	123	3	gain	***DLK1*** *, MIR2392, MEG3.*
		22q11.21	21298060	21407086	66	109	3	gain	*CRKL, LINC01637, **AIFM3**, LZTR1, THAP7, TUBA3FP, P2RX6, SLC7A4, MIR649, P2RX6P, LRRC74B.*

* Medullary thyroid carcinoma samples in which CNA was identified. ** hg19. *** CNA found near the centromere. In bold, the selected genes used for validation analysis. CN, copy number.

## Data Availability

The data presented in this study are available on request from the corresponding author. The data are not publicly available as the data repository is under construction.

## Supplementary Materials

The following are available online at https://www.mdpi.com/2072-6694/13/2/218/s1, Table S1: Distribution of copy number losses and gains in the discovery cohort. Table S2: Immunohistochemistry staining level in twenty-seven MTC samples. Table S3: Copy number alteration (CNA) in *AIFM3* and *DLK1* described on Catalogue of Somatic Mutations in Cancer (COSMIC). Table S4: Clinical–pathological features of discovery cohort patients. Table S5: Clinical–pathological features of the Independent set of MTC cases used for experimental validation analysis. Table S6: Primer used for PCR amplification and expected product size.
