# Supplementary material for: Comprehensive Assessment of Copy Number Alterations Uncovers Recurrent AIFM3 and DLK1 Copy Gain in Medullary Thyroid Carcinoma
Source: Cancers (Basel). 2021 Jan 9;13(2):218. doi: 10.3390/cancers13020218 (PMC7826827; doi:10.3390/cancers13020218)
Supplement: Supplementary file 1 [file cancers-13-00218-s001.zip › Araujo_et_al._Supplementary_Table_2.docx]

**Table S2:** Immunohistochemistry staining level in twenty-seven MTC samples.

| **Patients** | **AIFM3** | | **DLK1** | |
| --- | --- | --- | --- | --- |
|  | **CN** | **IHC** | **CN** | **IHC** |
| 1 | 4 | ` | 4 | +++ |
| 3 | 4 | +++ | 4 | +++ |
| 7 | 4 | +++ | 4 | ++ |
| 8 | 4 | +++ | 3 | ++ |
| 9 | 4 | + | 4 | ++ |
| 12 | 4 | ++ | 4 | +++ |
| 16 | 4 | ++ | 4 | +++ |
| 17 | 4 | +++ | 4 | +++ |
| 18 | 4 | ++ | 4 | +++ |
| 24 | 3 | - | 2 | - |
| 25 | 2 | ++ | 2 | ++ |
| 26 | 4 | - | 2 | - |
| 27 | 4 | +++ | 4 | +++ |
| 28 | 4 | +++ | 4 | ++ |
| 29 | 4 | +++ | 4 | +++ |
| 31 | 4 | +++ | 4 | +++ |
| 32 | 4 | ++ | 4 | ++ |
| 33 | 4 | ++ | 4 | +++ |
| 34 | 4 | ++ | 3 | + |
| 36 | 4 | ++ | 4 | +++ |
| 37 | 4 | ++ | 4 | ++ |
| 38 | 3 | +++ | 4 | ++ |
| 39 | 4 | - | 4 | + |
| 40 | 4 | +++ | 4 | +++ |
| 45 | 4 | ++ | 4 | +++ |
| 48 | 4 | ++ | 4 | ++ |
| 51 | 3 | +++ | 4 | +++ |

**CN:** Copy Number state. **ICH:** Immunohistochemistry (staining level: + low, ++ medium, +++ high).
