# Supplementary material for: Comprehensive Assessment of Copy Number Alterations Uncovers Recurrent AIFM3 and DLK1 Copy Gain in Medullary Thyroid Carcinoma
Source: Cancers (Basel). 2021 Jan 9;13(2):218. doi: 10.3390/cancers13020218 (PMC7826827; doi:10.3390/cancers13020218)
Supplement: Supplementary file 1 [file cancers-13-00218-s001.zip › Araujo_et_al._Supplementary_Table_3.docx]

**Table S3:** Copy Number Alteration (CNA) in *AIFM3* and *DLK1* described on Catalogue Of Somatic Mutations in Cancer COSMIC.

| **Cancer Tissue** | **AIFM3** | | | | | | **DLK1** | | | | | |
| --- | --- | --- | --- | --- | --- | --- | --- | --- | --- | --- | --- | --- |
|  | **CNA** | | | **Gene Expression** | | | **CNA** | | | **Gene Expression** | | |
|  | **Losses** | **Gains** | **Tested** | **No/Low** | **Over** | **Tested** | **Losses** | **Gains** | **Tested** | **No/Low** | **Over** | **Tested** |
| Adrenal gland | 2 | 0 | 268 | 0 | 1 | 79 | 1 | 0 | 268 | 0 | 27 | 79 |
| Breast | 4 | 2 | 1544 | 0 | 49 | 1104 | 3 | 3 | 1544 | 0 | 15 | 1104 |
| Central nervous system | 0 | 1 | 1093 | 0 | 28 | 697 | 1 | 0 | 1093 | 0 | 11 | 697 |
| Cervix | - | - | - | 0 | 19 | 307 | 0 | 1 | 313 | 0 | 4 | 307 |
| Endometrium | - | - | - | 0 | 36 | 602 | 0 | 1 | 598 | 0 | 14 | 602 |
| Esophagus | 1 | 3 | 499 | - | - | - | 0 | 2 | 499 | 0 | 1 | 125 |
| Hematopoietic and lymphoid | 0 | 1 | 819 | 0 | 10 | 221 | 0 | 1 | 819 | 0 | 7 | 221 |
| Kidney | 0 | 1 | 1027 | 0 | 20 | 600 | 4 | 0 | 1027 | 0 | 11 | 600 |
| Large intestine | 1 | 0 | 771 | 0 | 17 | 610 | - | - | - | 0 | 27 | 610 |
| Liver | - | - | - | 0 | 33 | 373 | 1 | 1 | 871 | 0 | 11 | 373 |
| Lung | 1 | 16 | 1185 | 0 | 77 | 1019 | 1 | 10 | 1185 | 0 | 13 | 1019 |
| Ovary | 0 | 3 | 729 | 0 | 11 | 266 | 0 | 2 | 729 | 0 | 13 | 266 |
| Pancreas | - | - | - | 0 | 15 | 179 | - | - | - | 0 | 9 | 179 |
| Pleura | 0 | 1 | 108 | - | - | - | - | - | - | - | - | - |
| Prostate | - | - | - | 0 | 18 | 498 | - | - | - | 0 | 10 | 498 |
| Skin | 0 | 8 | 630 | 0 | 34 | 473 | 1 | 0 | 630 | 0 | 7 | 473 |
| Soft tissue | - | - | - | 0 | 11 | 263 | 0 | 1 | 276 | 0 | 17 | 263 |
| Stomach | 0 | 1 | 501 | - | - | - | - | - | - | 0 | 14 | 285 |
| Testis | 0 | 2 | 152 | - | - | - | 0 | 1 | 152 | - | - | - |
| Thymus | 0 | 1 | 124 | - | - | - | - | - | - | - | - | - |
| Thyroid | - | - | - | 0 | 23 | 513 | - | - | - | 0 | 3 | 513 |
| Upper aerodigestive tract | 1 | 7 | 563 | 0 | 44 | 522 | 0 | 4 | 563 | 0 | 14 | 522 |
| Urinary tract | 0 | 8 | 419 | 0 | 8 | 408 | 1 | 0 | 419 | 0 | 1 | 408 |
| **Total** | 10 | 55 | 10432 | 0 | 454 | 8734 | 13 | 27 | 10986 | 0 | 229 | 9144 |
