# Supplementary material for: Comprehensive Assessment of Copy Number Alterations Uncovers Recurrent AIFM3 and DLK1 Copy Gain in Medullary Thyroid Carcinoma
Source: Cancers (Basel). 2021 Jan 9;13(2):218. doi: 10.3390/cancers13020218 (PMC7826827; doi:10.3390/cancers13020218)
Supplement: Supplementary file 1 [file cancers-13-00218-s001.zip › Araujo_et_al._Supplementary_Table_1.doc]

**Table S1: Distribution of copy number losses and gains in the discovery cohort**

| **Software** | **Sample** | **Number of Losses** | **Mean length of Losses (bp)** | **Number of Gains** | **Mean length of Gains (bp)** | **Total Number of CNAs** | **Mean length of CNAs (bp)** |
| --- | --- | --- | --- | --- | --- | --- | --- |
| **Penn** | 1 | 32 | 27873 | 9 | 105329 | 41 | 44876 |
|  | 2 | 12 | 16869 | 20 | 78522 | 32 | 55402 |
|  | 3 | 0 | 0 | 8 | 29438 | 8 | 29438 |
|  | Total | 44 | 44742 | 37 | 213289 | 81 | 129716 |
|  | Mean | 15 | 14914 (15kb) | 12 | 71096 (71kb) | 27 | 43239 (43kb) |
|  |  |  |  |  |  |  |  |
| **GTC** | 1 | 9 | 140110 | 17 | 535075 | 26 | 392888 |
|  | 2 | 189 | 162806 | 31 | 144050 | 220 | 160163 |
|  | 3 | 2 | 144752 | 17 | 301090 | 19 | 284633 |
|  | Total | 200 | 447668 | 66 | 980215 | 266 | 837684 |
|  | Mean | 67 | 149223 (149kb) | 22 | 326738 (327kb) | 89 | 279228 (279kb) |
