# Supplementary material for: Comprehensive Assessment of Copy Number Alterations Uncovers Recurrent AIFM3 and DLK1 Copy Gain in Medullary Thyroid Carcinoma
Source: Cancers (Basel). 2021 Jan 9;13(2):218. doi: 10.3390/cancers13020218 (PMC7826827; doi:10.3390/cancers13020218)
Supplement: Supplementary file 1 [file cancers-13-00218-s001.zip › Araujo_et_al._Supplementary_Table_4.doc]

**Table S4: Clinical-pathological features of discovery cohort patients**

| **Sample** | **Age at Diagnosis (yr) / Gender** | **RET Germline Mutation** | **Tumor Size (cm)** | **pTNM** | **PHEO** |
| --- | --- | --- | --- | --- | --- |
| T1 | 47/M | G533C | 2.5 | T2N1Mx | No |
| T2 | 14/M | M918T | 1.2 | T4N1Mx | No |
| T3 | 21/M | M918T | 1.5 | T4N1Mx | Yes |
|  | | | | | |
