# Supplementary material for: Comprehensive Assessment of Copy Number Alterations Uncovers Recurrent AIFM3 and DLK1 Copy Gain in Medullary Thyroid Carcinoma
Source: Cancers (Basel). 2021 Jan 9;13(2):218. doi: 10.3390/cancers13020218 (PMC7826827; doi:10.3390/cancers13020218)
Supplement: Supplementary file 1 [file cancers-13-00218-s001.zip › Araujo_et_al._Supplementary_Table_5.doc]

**Table S5: clinical-pathological features of the Independent set of MTC cases used for experimental validation analysis.**

| **Patients** | **Age at Diagnosis (yr) /Gender** | **Tumor Type** | **Driver Mutation** | **Tumor Size (cm)** | **Multifocality** | **Extrathyroidal extension** | **Vascular invasion** | **pTNM** | **AJCC** |
| --- | --- | --- | --- | --- | --- | --- | --- | --- | --- |
| 1 | 71/F | S | NA | 8 | NA | NA | Yes | T3N1bMx | IVA |
| 2 | 44/M | H | RET C634R | 4.2 | NA | NA | NA | T3N1bMx | IVA |
| 3 | 59/F | S | No | 1.6 | Yes | No | No | T1N1bMx | IVA |
| 4 | 32/F | S | No | 2.4 | No | No | No | T2N0Mx | II |
| 5 | 56/M | S | NA | 1 | Yes | Yes | Yes | T3N1bMx | IVA |
| 6 | 48/F | S | NA | 2.3 | Yes | No | No | T2NxMx | II |
| 7 | 58/F | S | No | 1.5 | NA | No | No | T1N0Mx | I |
| 8 | 69/M | S | HRAS Q61R | 2.7 | No | No | Yes | T3N0Mx | II |
| 9 | 38/M | S | No | 2.2 | No | No | NA | T2N1aMx | III |
| 10 | 65/F | S | NA | NA | Yes | No | No | NA | NA |
| 11 | 43/M | H | RET C634Y/Y791F | 2.9 | Yes | No | Yes | T2N0Mx | II |
| 12 | 41/F | H | RET C634 | 5 | Yes | NA | NA | T3N1bMx | IVA |
| 13 | 39/M | H | RET G533C | 0.5 | Yes | No | No | T1N0Mx | I |
| 14 | 42/M | H | RET G533C | 0.2 | Yes | No | No | T1N0Mx | I |
| 15 | 21/M | H | RET G533C | 0.9 | No | No | Yes | T1N0Mx | I |
| 16 | 33/M | H | RET C634Y/Y791F | 0.8 | Yes | No | No | T1NxMx | I |
| 17 | 19/F | H | RET C634Y/Y791F | 1.2 | Yes | NA | NA | T1N1aMx | III |
| 18 | 21/M | H | RET C634Y/Y791F | 1.2 | Yes | No | Yes | T1N1aMx | III |
| 19 | 61/M | H | RET G533C | 2.3 | Yes | No | No | T2N1aMx | III |
| 20 | 24/F | H | RET G533C | 0.4 | NA | NA | NA | T1NxM0 | I |
| 21 | 43/M | H | RET G533C | 0.9 | Yes | No | No | NA | NA |
| 22 | 26/M | H | RET C634Y/Y791F | 1.5 | Yes | No | NA | T1N1aMx | III |
| 23 | 51/F | H | RET C634 | 5.5 | Yes | NA | Yes | T3N1bMx | IVA |
| 24 | 72/M | H | RET C634Y | 0.8 | Yes | No | No | T1NxMx | I |
| 25 | 23/F | H | RET C634Y | 2.2 | No | No | No | T2NxMx | II |
| 26 | 45/M | H | RET C634Y | 1 | Yes | No | No | T1N1bMx | IVA |
| 27 | 25/F | S | NA | 4 | NA | No | Yes | T2N1bMx | IVA |
| 28 | 55/F | S | No | 2.5 | Yes | Yes | Yes | T3N1bMx | IVA |
| 29 | 27/M | S | RET M918T | NA | Yes | Yes | Yes | T4aN1bMx | IVA |
| 30 | 49/F | S | NA | 5 | No | No | No | T3NxMx | II |
| 31 | 65/F | S | NA | 2 | No | No | Yes | T1N1aMx | III |
| 32 | 69/M | S | NA | 4 | No | No | Yes | T2N0Mx | II |
| 33 | 67/M | S | No | 2.9 | No | No | Yes | T2N0Mx | II |
| 34 | 52/M | S | RET C634Y | 2.9 | No | No | Yes | T2NxMx | II |
| 35 | 50/F | S | RET M918T | NA | NA | NA | NA | T3N1bMx | IVA |
| 36 | 37/M | S | No | 5 | No | No | No | T2NxMx | II |
| 37 | 62/F | S | HRAS Q61R | 3.7 | No | No | No | T2N0Mx | II |
| 38 | 43/F | S | No | 0.9 | No | No | No | T1N0Mx | I |
| 39 | 51/M | S | RET M918T | 3.2 | Yes | Yes | Yes | T3N1bMx | IVA |
| 40 | 60/F | S | RET M918T | 4 | No | No | No | T2N0Mx | II |
| 41 | 63/F | S | NA | 0.3 | NA | NA | NA | T1NxMx | I |
| 42 | 51/F | S | NA | 3.5 | No | No | No | T2N0Mx | II |
| 43 | 64/F | S | NA | 2 | No | No | No | T1N0Mx | I |
| 44 | 49/F | H | C634R | 1.7 | Yes | No | NA | T1N1aMx | III |
| 45 | 43/F | H | C609W | 2.7 | Yes | No | NA | T2NxMx | II |
| 46 | 33/F | S | NA | 2.6 | No | No | Yes | T2N0Mx | II |
| 47 | 37/F | S | RET Y701F | 2.5 | NA | No | Yes | T2N1aMx | III |
| 48 | 48/F | H | RET C634Y/Y791F | 1.8 | Yes | Yes | Yes | T3N1bMx | IVA |
| 49 | 83/F | S | NA | 2.3 | No | No | Yes | T2N1bMx | IVA |
| 50 | 70/F | S | NA | 0.8 | No | No | No | T1N1bMx | IVA |
| 51 | 47/F | S | NA | 1.3 | No | No | No | T1N0Mx | I |

S: sporadic; H: hereditary; NA: Not available; EE: Extrathyroidal Extension; VI: Vascular Invasion
