# Supplementary material for: Comprehensive Assessment of Copy Number Alterations Uncovers Recurrent AIFM3 and DLK1 Copy Gain in Medullary Thyroid Carcinoma
Source: Cancers (Basel). 2021 Jan 9;13(2):218. doi: 10.3390/cancers13020218 (PMC7826827; doi:10.3390/cancers13020218)
Supplement: Supplementary file 1 [file cancers-13-00218-s001.zip › Araujo_et_al._Supplementary_Table_6.doc]

**Table S6:** Primer used for PCR amplification and expected product size

|  | **Locus/Gene** | **Primer Sequence** | **Product Size** |
| --- | --- | --- | --- |
| CNA validation (qPCR) | ***ADAM8*** | F: 5'- CCCCAGCCCTGTCCTTAC -3' | 102 |
|  |  | R: 5'- GCTCTGGGACAGCCTTAGC -3' |  |
|  | ***AIFM3*** | F: 5'- TGGAACACGGAGCAGTGGC -3' | 111 |
|  |  | R: 5'- CTTCTCCCTCCTACAATGGG -3' |  |
|  | ***DLK1*** | F: 5'- CCAGTGCGTGACCTCTCC -3' | 107 |
|  |  | R: 5'- AGTGCCAACCTCTATCACAG -3' |  |
|  | ***KGFLP2*** | F: 5'- ACGGAGGGGAAATGTTTGTG -3' | 144 |
|  |  | R: 5'- CTGCTGGAACTGGTTCTTTAT -3' |  |
|  | ***SNORD116-18*** | F: 5'- GGTGCTAGTGGATTCCTTAG -3' | 146 |
|  |  | R: 5'- GTTTTCACTCATTTTGTTCAGC -3' |  |
|  | **4q28.3** | F: 5'- GGTATTCACCCTTATGTATGG -3' | 115 |
|  |  | R: 5'- GGTTTGTTCCTCTTAGCCAG -3' |  |
|  | ***ACTB*** | F: 5’-CCCTTCCCTCCTCAGATCATT -3’ | 108 |
|  |  | R: 5'- CTTGCTGATCCACATCTGCTG -3' |  |
